# Response to Imatinib therapy is inferior for e13a2 *BCR-ABL1* transcript type in comparison to e14a2 transcript type in chronic myeloid leukaemia

**DOI:** 10.1186/s12878-019-0139-2

**Published:** 2019-05-02

**Authors:** Graeme Greenfield, Ross McMullan, Nuala Robson, Julie McGimpsey, Mark Catherwood, Mary Frances McMullin

**Affiliations:** 1 Centre for Cancer Research and Cell Biology, Queen’s University Belfast, 97 Lisburn Rd, Belfast, BT9 7NN UK; 2 Department of Haematology, Belfast City Hospital, Belfast, UK; 3 CME, Queen’s University Belfast, Belfast, UK

**Keywords:** Chronic myeloid Leukaemia, Treatment free remission, *BCR-ABL*, e13a2 transcript, e14a2 transcript

## Abstract

**Background:**

The *BCR-ABL1* fusion gene underlying the pathogenesis of CML can arise from a variety of breakpoints. The e13a2 and e14a2 transcripts formed by breakpoints occurring around exon 13 and exon 14 of the *BCR* gene respectively are the most common.

**Methods:**

We undertook a retrospective audit using local laboratory database and electronic patient care records of 69 CML patients with an e13a2 or e14a2 transcript type identified in our regional population.

**Results:**

The e13a2 group was on average significantly younger (45.0 years v 54.5 years), had a higher average white cell count (189.8 × 10^9^/l v 92.40 × 10^9^/l) and lower platelet count (308 × 10^9^/l v 644 × 10^9^/l) in comparison to the e14a2 group suggesting that these are distinct biological entities. Over an average follow-up of 33.8 months and 27.2 months for the e13a2 and e14a2 groups we observed an inferior molecular response to imatinib in the e13a2 group. A significantly lower number of patients in the e13a2 arm met European Leukemia Net criteria for optimal response at 12 months therapy (17.64% v 50.0%) and were slower to obtain deep molecular responses MR^4^ or MR^4.5^.

**Conclusion:**

Patients with an e13a2 transcript demonstrate an inferior molecular response to imatinib in our regional population.

## Background

Chronic myeloid leukaemia (CML) is characterised in virtually all cases by the presence of a translocation between chromosomes 9 and 22 resulting in the formation of the Philadelphia chromosome and a *BCR-ABL1* fusion protein [1–4]. This fusion results in the formation of a constitutively active tyrosine kinase driving proliferation of the myeloid lineage producing the disease phenotype [5]. Diagnosis of CML is based on characteristic blood findings co-existent with the presence of the Philadelphia chromosome or detection of *BCR-ABL1* fusion gene by polymerase chain reaction (PCR) or fluorescent in situ hybridisation (FISH) [6].

Within the last 20 years, the development of specific tyrosine kinase inhibitors (TKIs) targeting the *BCR-ABL1* fusion protein has revolutionised treatment of the disease producing deep and sustained haematological and molecular responses [7, 8]. Imatinib is the first generation of these TKIs and is the most frequently used TKI as first line therapy in our setting. Treatment free remissions following a sustained period of imatinib therapy are now regularly reported [9]. However, other patients respond less well to imatinib therapy. In some cases, there is a failure to obtain an adequate molecular response, progression of disease to accelerated or blast phase or loss of previously obtained molecular responses. This can represent clonal evolution with acquisition of a mutation in the genomic sequence encoding the *BCR-ABL1* transcript in some instances [10].

It is well recognised that there is variation in the breakpoints that can occur to allow formation of *BCR-ABL1* fusion transcript [11]. In the vast majority of cases this results in the formation of 210 kDa tyrosine kinase (p210) with a smaller number producing a 190 kDa or 230 kDa product. The p210 BCR-ABL1 may be encoded by a number of different transcripts. The most common of these are e13a2 (also notated b2a2) and e14a2 (also notated b3a2) accounting for greater than 95% of the CML population [11]. The e13a2 is formed from a breakpoint at the 5′ aspect of the *BCR* gene around exon 13 fused to exon 2 of the *ABL1* gene. The e14a2 results from a breakpoint in the 3′ aspect of the *BCR* gene around exon 14 again fused to exon 2 of the *ABL* gene. This results in a difference of 75 base pairs in the hybrid mRNA between the two sequences and therefore a difference of 25 amino acids in the resulting BCR-ABL1 fusion protein [12]. Alternative splicing mechanisms mean that in patients with the e14a2 transcript, either the e13a2 or e14a2 may be expressed from the one clone [13].

The relevant prognostic value of the underlying transcript type was evaluated in the pre TKI era but without conclusive evidence of significant difference established [12]. One study suggested that the duration of chronic phase and length of time to progression to blast disease was much shorter in the e14a2 group in comparison to the e13a2 group [14]. Other studies failed to substantiate this finding [15]. In the TKI era a number of studies have evaluated the prognostic value of underlying transcript type. A recent meta-analysis was suggestive of an inferior response in the e13a2 group [16]. There are also recent reports of a difference in the maintenance of treatment free remission dependent on transcript type [17].

On the basis of these findings, we set out to establish if the underlying transcript type was relevant for prognosis in our regional population for patients treated with imatinib first line with a focus on achievement of a deep molecular response.

## Methods

Our laboratory database of all positive diagnostic *BCR-ABL1* transcripts within the Northern Ireland region from 14/11/2011 was reviewed. Patients were excluded from further analysis if the diagnosis was not chronic myeloid leukaemia, if less than three months had passed since diagnosis or if the transcript type was not known. A retrospective audit of therapy and clinical outcomes was then undertaken for seventy-four patients (*n* = 74). Regional electronic patient records and local laboratory database were used to obtain data. Exclusions from further analysis were as follows; One patient with an atypical transcript type, three patients as were not initially commenced on imatinib therapy and one patient who presented in blast phase of disease as per WHO criteria. This left sixty-nine patients for analysis (*n* = 69).

Real-time quantitative PCR (RQ-PCR) was performed using RNA extracted from Peripheral blood for monitoring BCR-ABL1 transcript levels reported on the International Scale (IS) [18]. The IS conversion factor was derived from a sample exchange scheme performed by Professor Nick Cross (Wessex Regional Genetics Laboratory). RT-PCR was performed to determine transcript type as previously described [19].

Statistical analysis was performed using Microsoft Excel Software. Unpaired, two tailed t test was used to determine significance of quantitative data whilst Chi-square test of fit was used for assessment of qualitative results. A multivariate regression analysis was performed using all variables available for assessment. Binary variables (transcript type, gender and meeting optimal criteria) were coded as 0 and 1 for this analysis. *P* values less than 0.05 were considered statistically significant. Kaplan-Meier analysis was undertaken to determine overall survival, event free survival and probability of maintaining MMR. A log rank test was used to generate *p* values for Kaplan-Meier curves.

## Results

### Baseline demographics

A total of sixty-nine patients were identified as suitable for inclusion in this retrospective analysis. The baseline demographics are presented in Table 1 with the e14a2 transcript more common. The e13a2 group was significantly younger on average than the e14a2 group. There was also a significantly higher white cell count (WCC) and lower platelet count in the e13a2 group. Dual expressers have been reported previously, we did not identify any in this cohort.

**Table 1:** A table showing the baseline demographics and initial treatment for each group

	e13a2	e14a2	*p* value
Number			
%	29%	71.0%	n/a
Exact	20	49	
Age (years)			
Mean	45.0	54.57	0.043
Median	46.0	59.0	
Range	4–81	5–80	
Sex			
Male	75.0%	55.1%	0.125
Female	25.0%	44.9%	
Follow-up (months)			
Mean	33.85	27.24	0.192
Median	37.5	23	
Range	1–70	3–76	
Haemoglobin (g/l)			
Mean	113.5	121.3	0.134
Range	86–148	64–158	
White Cell Count			
(×10^9/^l)	189.80	92.40	0.0002
Mean	49.1–563	4.86–292	
Range			
Platelets (×10^9/^l)			
Mean	308	644	0.001
Range	93–1058	178–2507	
EUTOS SCORE			
Number available	15	42	0.685
Average Score	35.93	39.66	
% High Risk	5.0%	6.1%	
*BCR-ABL1* PCR level			
Number available	17	42	0.354
Mean	58.64%	71.0%	
Range	25.6–112	8.1–344	
Initial Treatment			
Imatinib 400 mg/day	90%	93.9%	n/a
Imatinib 300 mg/day	5%	0%	
Imatinib 200 mg/day	5%	6.1%	

### Effect of transcript type on molecular response

We evaluated the molecular response at 3 months (*n* = 56 pts), 6 months (*n* = 51pts) and 12 months (*n* = 47 pts). *BCR-ABL1* transcript levels within 1 calendar month either side of time points were deemed acceptable for inclusion. These levels were then compared against the European Leukaemia Net (ELN) criteria for optimal response at each time point [20].

**Fig. 1:**
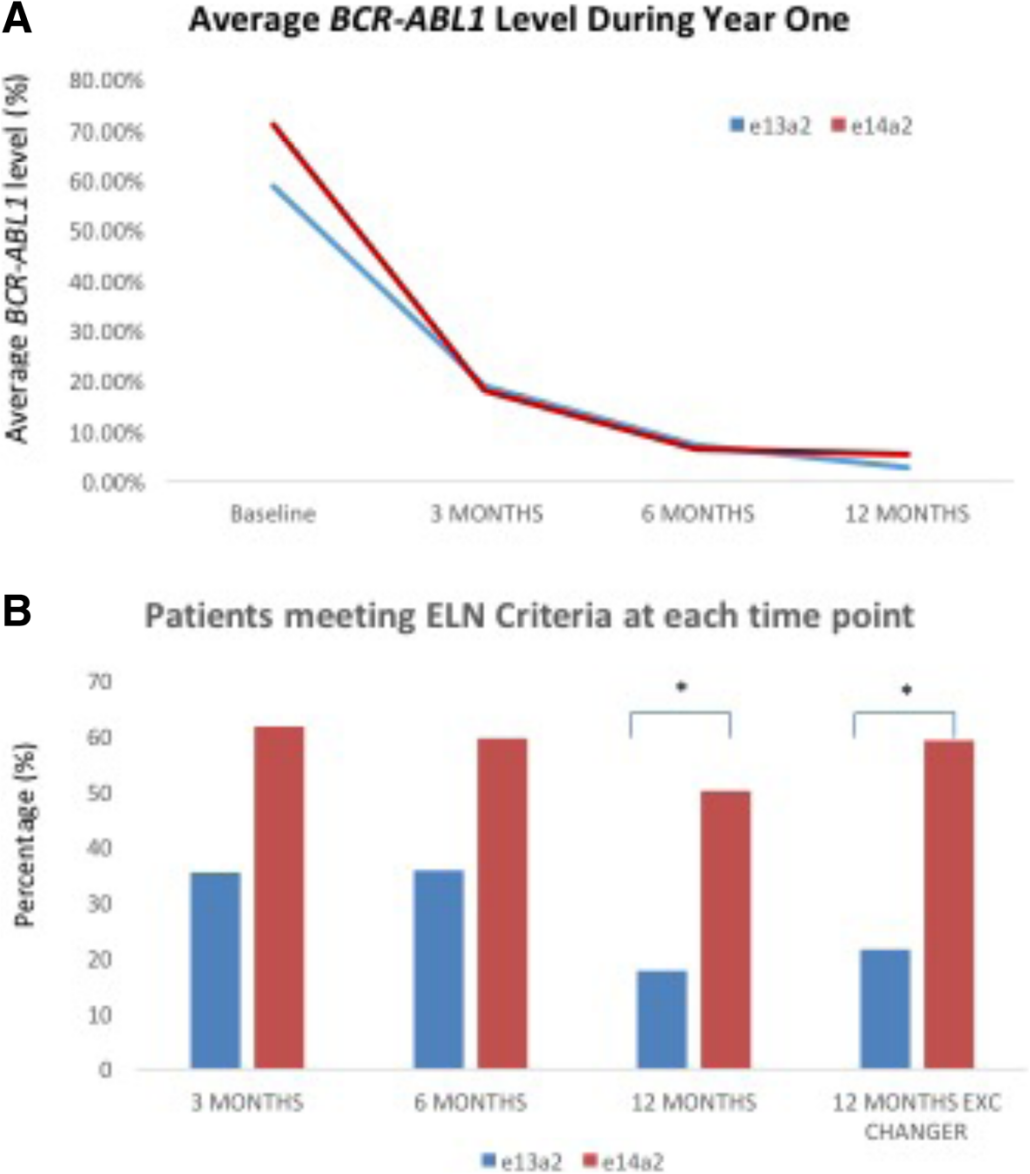
**a**) The trend of quantitative *BCR-ABL1* transcript levels by PCR over initial 12 months of therapy by transcript type. **b**) The number of patients meeting optimal ELN criteria by transcript type at each timepoint in year 1. EXC CHANGER = excludes patients changing TKI therapy prior to 12 months * = *p* value < 0.05

Figure 1a demonstrates the average *BCR-ABL1* transcript levels detected at each time point in year 1 for each group. As is evident there is no significant difference between the two groups at any time point. At 3 months, average *BCR-ABL1* levels were 18.93% in the e13a2 group and 18.08% in the e14a2 group. At 6 months this was 7.23 and 6.44% respectively and at 12 months 2.71 and 5.31% respectively. When the groups were compared against the ELN criteria, at 3 months and 6 months there was a higher percentage of patients in the e14a2 group meeting optimal response criteria. By 12 months this had reached statistical significance with fewer patients in the e13a2 group (17.64%) meeting ELN criteria for optimal response in comparison to the e14a2 group (50.0%). This is demonstrated in Fig. 1b. This was despite the non-significant difference observed in the quantitative assessment of *BCR-ABL1* transcript levels. When patients who had undergone a change of TKI therapy to second line therapy prior to 12 months were excluded from the analysis this statistical significance remained with 21.2% of e13a2 patients meeting criteria compared with 59.1% of e14a2 patients. Again there was no significant difference observed in the quantitative *BCR-ABL1* transcript level when changers prior to 12 months were excluded (e13a2: 1.75%, e14a2: 1.64%).

**Fig. 2:**
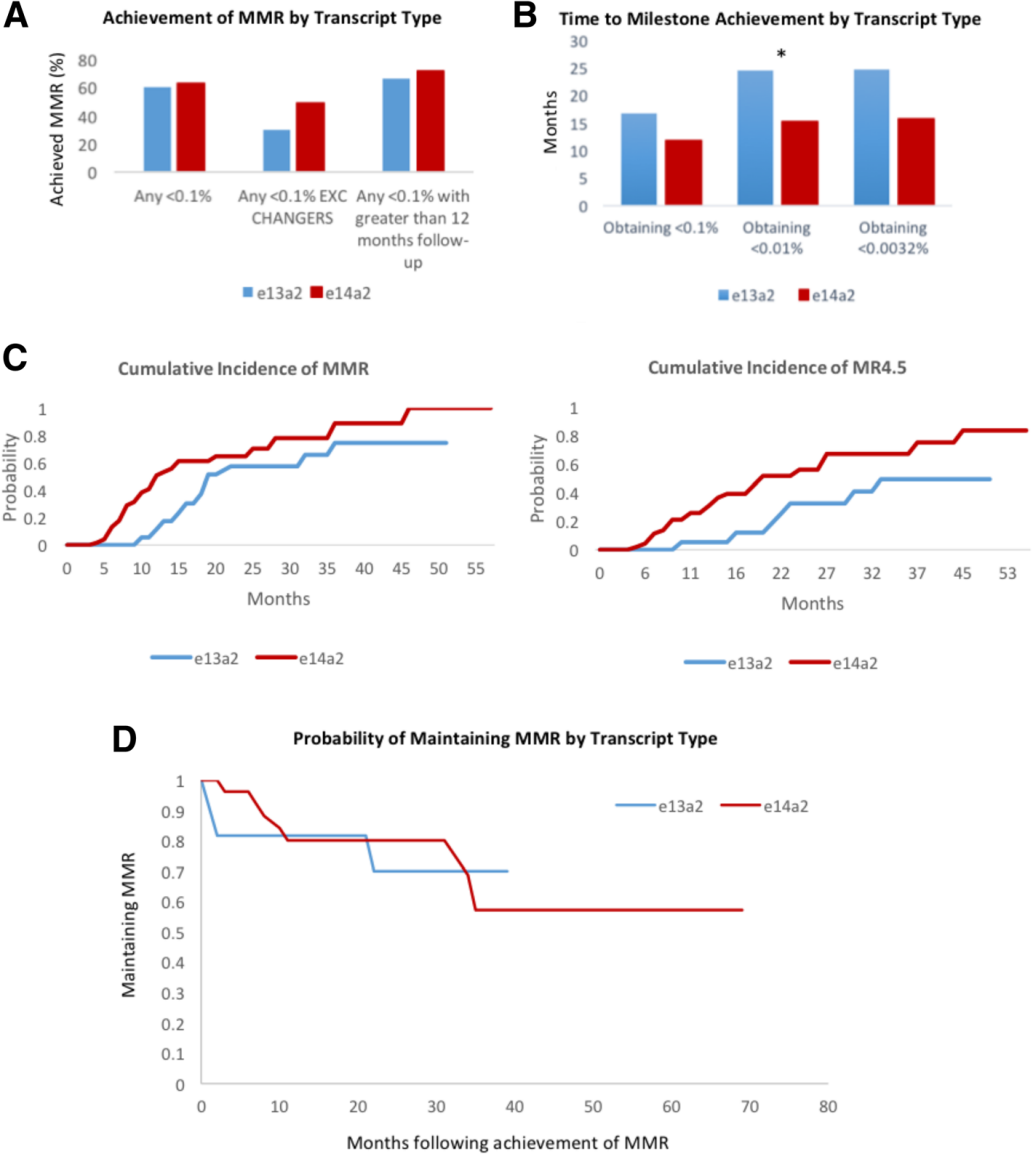
**a**) A chart showing the number of patients obtaining a MMR (*BCR-ABL1* < 0.1%) by transcript type for all patients, excluding patients with change of TKI therapy prior to milestone and for patients with greater than 12 months follow-up. **b**) Time to achievement of MMR (*BCR-ABL1* < 0.1%), MR^4^ (*BCR-ABL1* < 0.01%) and MR^4.5^ (*BCR-ABL1* < 0.0032%) by transcript group. * = *p* < 0.05. **c**) The cumulative incidence of obtaining MMR and MR^4.5^ are demonstrated using a 1-KM method. **d**) Probability of maintaining MMR once achieved over time of follow-up by transcript type, calculated using Kaplan Meier methodology. *p* = 0.08 using log rank test

Obtaining a major molecular response (MMR) is a primary objective in the therapy of CML within 12 months. This is defined as a *BCR-ABL1* transcript level less than 0.1% on the International Scale by ELN [20]. The number of patients obtaining a MMR at any stage during follow-up was assessed for both groups. The number of patients obtaining MMR was greater in the e14a2 arm for all patients (e13a2:60.0%, e14a2:63.2%), when patients undergoing change of therapy prior to obtaining MMR were excluded (e13a2: 30.0%, e14a2:48.9%), and in patients with greater than 12 months of follow-up available (e13a2: 66.6%, e14a2:71.7%). However, none of these differences reached statistical significance. This is demonstrated in Fig. 2a. When the time to obtain MMR was evaluated there was a trend towards earlier achievement of MMR in the e14a2 group for all patients and excluding changers which again did not reach significance (e13a2: 16.75 months and 11.5 months, e14a2: 11.80 months and 9.58 months). We then examined deeper molecular responses. Achievement of MR^4^ (*BCR-ABL1 <* 0.01%) is crucial for the purposes of attempting treatment free strategies. We evaluated the number of patients obtaining levels less than 0.01% and found no significant difference (e13a2: 40.0%, e14a2: 51.0%) between the two groups. However, there was a significant improvement in time to achieve this seen in the e14a2 group (e13a2: 24.5 months, e14a2: 15.6 months). The results were similar for achieving MR^4.5^ with *BCR-ABL1* < 0.0032% however, the difference between the two groups was marginally outside the level of significance for time to achievement (*p* = 0.05). This is shown in Fig. 2b. Figure 2c shows the cumulative incidence of obtaining MMR and MR^4.5^ by transcript type using 1-KM methodology. This demonstrates a superior response for e14a2 transcripts which was significant (*p* < 0.05) by Wilcoxon-Gehan methodology and borderline for significance by log rank test (MMR *p* = 0.10 and MR^4.5^ *p* = 0.07).

Loss of MMR is suggestive of treatment failure or disease progression but could represent poor compliance with treatment. We defined loss of MMR as a sustained increase above 0.1% transcript level or a significant increase above 1%. There was no significant difference evident between the two groups on comparison of the percentage of patients losing MMR having previously achieved it at any stage. (e13a2: 25.0%, e14a2: 22.5%). Fig. 2d demonstrates there was no difference in the probability of maintaining MMR for each transcript. (*p* = 0.08). In 70% of cases of loss of MMR this was a transient phenomenon.

A multivariate regression analysis was then undertaken to establish if other variables were significant in obtaining an optimal ELN criteria response of less than 0.1% at 12 months. Independent variables included in this analysis were transcript type, age, gender, EUTOS score at diagnosis, *BCR-ABL1* level at baseline, baseline haemoglobin, baseline white cell count and baseline platelet count. Patients without a 12 month *BCR-ABL1* result were excluded. As not all variables were available for every patient, four multivariate analyses were then run to include patients with all variables (*n* = 29), patients with all variables excluding EUTOS score (*n* = 39), patients with all variables excluding *BCR-ABL1* level (*n* = 35) and patients with all variables excluding EUTOS and *BCR-ABL1* levels. The significance of transcript type in determining optimal response at 12 months was lost in each of these analyses. No other variables were consistently significant across the four analyses.

### The effect of transcript type on therapy

**Fig. 3:**
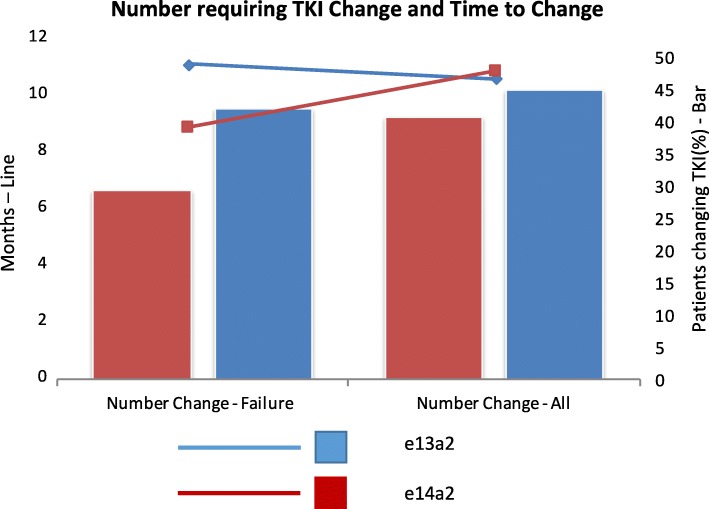
A bar chart showing the number of patients changing TKI by transcript type for all reasons and as a result of treatment failure. Superimposed on this is a line chart showing time to change for each transcript type for all reasons and for treatment failure

The local laboratory system does not routinely release the transcript type. Therefore, treating physicians across the region are effectively blinded to this information when making decisions to change therapy. We first assessed the number of patients in each group receiving a change of TKI therapy for all reasons (treatment failure, intolerance or unacceptable toxicity and patient choice). There was no significant difference between the number of patients in either group moving to second line therapy (e13a2: 45%, e14a2: 40.8%) or significant difference between the average time to switch (e13a2: 11 months, e14a2: 8.85 months). When we examined those switching on account of treatment failure only, the results were similar for numbers (e13a2 40%, e14a2: 24.4%) or for time to switch (e13a2: 10.5 months, e14a2: 10.75 months). Fig. 3 demonstrates this.

### Clinical outcomes by transcript type

**Fig. 4:**
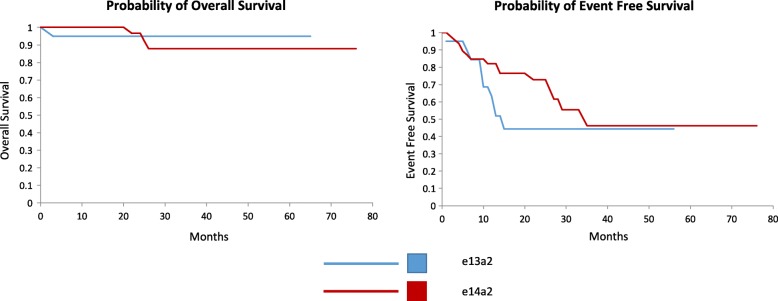
Graphs showing Kaplan-Meier analysis of overall and event free survival by transcript type. Event free survival defined as survival in the absence of death, blast crisis, failure of therapy requiring change or loss of MMR. Intolerance of therapy was not included

Finally, we examined the clinical outcomes by transcript type. Figure 4 shows the overall survival (OS) and event free survival of both groups (EFS) showing no significant difference between the two groups. During our follow-up we observed two transformations to blast crisis both within the e14a2 group. We also excluded one patient from analysis at the outset presenting with blast crisis by WHO criteria who was also an e14a2 transcript. Four deaths during follow-up were observed. Causes of death were documented as acute liver failure from imatinib therapy at 1 month follow-up, infective complications at 22 months, congestive cardiac failure at 26 months and intracranial haemorrhage secondary to blast crisis at 25 months. Allogeneic haematopoietic stem cell transplantation (HSCT) was undertaken in three patients, two post blast crisis and one for progressive disease not responding adequately to TKI therapy. All patients undergoing HSCT had e14a2 transcripts. Only 1 case of a mutation in the *BCR-ABL1* kinase domain was detected in our population. This patient went on to blast crisis.

Only two patients had stopped TKI therapy at the cessation of follow-up. Neither of these were as a result of elective stopping on account of meeting treatment cessation guidelines. Therefore, we are unable to comment on the impact of transcript type on maintenance of treatment free remission.

## Discussion

The role of front line imatinib therapy has been well established in the treatment of CML, Now patients with CML can expect an overall survival which is approaching that of the general population [21]. Therefore the goal of therapy in CML is shifting at the outset. Particularly in the younger patient, the aim is to achieve a early and sustained deep molecular response. This opens the possibility of treatment free remission. European Society of Medical Oncology (ESMO) guidelines have defined criteria allowing physicians and patients to consider treatment withdrawal if response to initial therapy is deemed adequate. These guidelines are strict, requiring sustained therapy for a minimum of 5 years, having achieving MR^4.5^ (*BCR-ABL1*^*IS*^ *< 0.0032%),* sustained MR^4^ (*BCR-ABL1*^*IS*^ *< 0.01%)* for over 2 years and optimal response on first line therapy in patients with a low SOKAL score at diagnosis and typical e13a2 or e14a2 transcript type [6]. Although the withdrawal of treatment is not going to be the favoured option for all patients, the range of second generation and beyond TKIs mean that as physicians the decisions regarding initial therapy will impact on patients options further down the line if there are slight differences in efficacy in particular circumstances. Therefore, obtaining an optimal response with first line therapy is of critical importance to patients wishing to regain the normality of treatment free life.

The e14a2 transcript type was more common in our population. The majority of other studies also report this although there may be some differences across varying ethnicities [16]. Our laboratory did not detect any co-expressers. The prevalence of these co-expressers has been between 0 and 20% in other cohorts therefore this may just be on the basis of the limited patient numbers in our study [16]. Our findings of a differential in WCC and platelet count point to a biological difference between these two groups. There is a bias towards thrombopoiesis in cells containing e14a2 and a bias towards leucopoiesis in those with e13a2. The difference in platelet count has been regularly reported previously in the majority or trials with the difference in white cell count also reported but less frequently [16, 22, 23]. There is a 75 base pair difference between the two transcripts resulting from the differing breakpoints. This results in a difference of 25 amino acid residues in the resulting fusion tyrosine kinase. Whether this difference in proliferative capacity is a direct result of the action of this tyrosine kinase mediating differences in downstream signal transduction pathways has not been demonstrated, however the secondary structure is predicted to differ between the two proteins [24]. It is conceivable that this structural difference may impact on TKI effectiveness.

Molecular responses evaluated by RT-PCR form a critical component of follow-up for CML, and directions on the continuation or alteration of specific therapies are based upon these. At low levels of disease burden, FISH and cytogenetics are insufficiently sensitive to detect CML clonal cells. We have not examined whether amplification of either allele is identical by RT-PCR in our individual setting. However, given that clinical decision making is based upon the IS *BCR-ABL1* levels derived from these RT-PCR levels this does not impact on the validity of the study. When molecular responses were examined, we identified a trend towards improved responses in the e14a2 group reaching statistical significance for the number of patients meeting optimal response by ELN criteria at 12 months and time to achieve a deep molecular response MR^4^. There was also a trend towards improved responses for achieving optimal response at 3 and 6 months, achieving MMR and MR^4.5^ at an earlier stage and a lower number of patients requiring therapy change due to treatment failure in the e14a2 group. Therefore, our results are consistent with improved molecular responses to front line imatinib therapy in a real world population of unselected individuals with CML for patients with the e14a2 group in comparison to the e13a2 group. Our study is limited by relatively small numbers but these results are largely in keeping with previous studies showing that patients with e14a2 obtain deeper, more sustained responses, faster than the e13a2 patients [16, 22, 23]. In our study and others, this does not impact on clinical outcomes [25]. A recent study comparing the expression of the *BCR-ABL1* transcript to the level of genomic *BCR-ABL1* DNA present in cells has suggested that although the expression of *BCR-ABL1* messenger RNA (mRNA) may be similar between the two groups, the level of genomic *BCR-ABL1* DNA is higher in e13a2 groups [26]. This may suggest that imatinib may be effective in reducing mRNA expression in both groups but less effective at reducing leukaemic cell burden in the e13a2 group. This may then underpin some of the differences we have observed when comparing deep molecular responses.

As OS and EFS were very similar between the two groups there is little evidence to suggest that in the current real world patient population that the type of transcript will ultimately affect the disease course. This, it could be argued, renders all of the above discussion as largely academic. However, we would suggest that given the critical need to obtain adequate deep and sustained molecular responses to allow patients the chance of treatment free life that there is an argument for the choice of TKIs with increased potency in the front line setting for younger patients with e13a2 transcripts. One study has suggested that the molecular response to imatinib in the e13a2 is inferior to that of second generation TKIs but that this was not seen in the e14a2 group [23]. Ideally, whether this front line use of second generation TKIs in this group will overcome some of the observed differences should be tested in a prospective study and may need to be offset against the greater toxicities of these agents. Ultimately this may help physician and patient make more informed decisions at diagnosis and allow for a more personalised approach to initial therapy. We did observe blast crisis only in the e14a2 group and the need for allogeneic stem cell transplantation only in the e14a2 group which appears contradictory to the other findings. Given the higher number of e14a2 patients neither of these were of statistical significance. One study had suggested a higher rate of blast crisis transformation in the e13a2 group [27].

## Conclusion

Our own approach over the past seven years has been homogenous with regards to front line therapy. With the critical importance of obtaining optimal early response to TKI therapy in allowing patients to attempt and sustain a treatment free remission, early personalised decisions regarding therapy may help to optimise treatment. Our results are in keeping with other published series in suggesting an inferior response in patients with an e13a2 transcript type. Prospective studies evaluating alternative TKI use in this group as up front therapy may overcome these findings in real world population studies may help to guide physician and patient decision making at the outset.

## Abbreviations

CML: Chronic Myeloid Leukaemia
EFS: Event Free Survival
ELN: European Leukaemia Net
ESMO: European Society of Medical Oncology
EUTOS: European Treatment and Outcome Study
FISH: Fluorescent in situ Hybridisation
HSCT: Haematopoietic Stem Cell Transplantation
IS: International Standard
MMR: Major Molecular Response
mRNA: Messenger RNA
OS: Overall survival
PCR: Polymerase Chain Reaction
RT-PCR: Real time Polymerase Chain Reaction
TKI: Tyrosine Kinase Inhibitor
WCC: White Cell Count
